# The associations between maternal lifestyles and antenatal stress and anxiety in Chinese pregnant women: A cross-sectional study

**DOI:** 10.1038/s41598-018-28974-x

**Published:** 2018-07-17

**Authors:** Qingzhi Hou, Shanshan Li, Chao Jiang, Yaling Huang, Lulu Huang, Juan Ye, Zhijian Pan, Tao Teng, Qiuyan Wang, Yonghua Jiang, Haiying Zhang, Chaoqun Liu, Mujun Li, Zengnan Mo, Xiaobo Yang

**Affiliations:** 1 Center for Genomic and Personalized Medicine, Guangxi Medical University, Nanning, Guangxi China; 2 Guangxi key Laboratory for Genomic and Personalized Medicine, Nanning, Guangxi China; 3 Guangxi Collaborative Innovation Center for Genomic and Personalized Medicine, Nanning, Guangxi China; 4 Guangxi Key Laboratory of Colleges and Universities, Nanning, Guangxi China; 5 Department of Occupational Health and Environmental Health, School of Public Health of Guangxi Medical University, Nanning, Guangxi China; 6 Department of Gynecology and Obstetrics, the Maternal & Child Health Hospital of Qinzhou, Qinzhou, Guangxi China; 7 Department of Antenatal care, the Maternal & Child Health Hospital of Nanning, Nanning, Guangxi China

## Abstract

The study aimed to investigate the associations between maternal lifestyles and antenatal stress and anxiety. 1491 pregnant women were drawn from the Guangxi birth cohort study (GBCS). A base line questionnaire was used to collect demographic information and maternal lifestyles. The Pregnancy Stress Rating Scale (PSRS) and Self-Rating Anxiety Scale (SAS) were used to assess prenatal stress and anxiety, respectively. Regression analyses identified the relationship between maternal lifestyles and prenatal stress and anxiety: (1) Hours of phone use per day was positively correlated to prenatal stress and anxiety and increased with stress and anxiety levels (all *P* trend < 0.05). In addition, not having baby at home was positively correlated to prenatal stress. (2) Self-reported sleep quality was negative with prenatal stress and anxiety, and decreased with stress and anxiety levels (all *P* trend < 0.01). Moreover, not frequent cooking was negatively correlated to prenatal stress and having pets was negatively correlated to prenatal anxiety (*P* < 0.05). However, having pets was not correlated to prenatal stress (*P* > 0.05). Our results showed that adverse lifestyles increase the risk of antenatal stress and anxiety, a regular routine and a variety of enjoyable activities decreases the risk of prenatal stress and anxiety.

## Introduction

Stress and anxiety are relatively common in pregnant women during the prenatal period^1–5^, this topic is currently receiving a large amount of attention from researchers. The immediate and longer-term consequences of antenatal stress and anxiety are far-reaching, not only affecting the mother but also her infant. Stress and anxiety during pregnancy could diminish one’s capacity for self-care, which could lead to inadequate nutrition, all of which could have influence on the gestation and delivery such as intrauterine growth restriction (IUGR)^6,7^, premature births^7–9^, low birth weight^9^ *et al*. Meanwhile, it could affect the nervous system development of infants and the psychological development of children^10^. Moreover, a recent study has revealed antenatal stress and anxiety could lead to postpartum psychological disorders and psychosis^11^. All of the above analyses highlight the importance of prenatal mental health care.

Improving care for prenatal mood disorders should depend on more effective risk factor prediction. Previous studies reported that sociodemographic factors, such as maternal age, marital status, education level^12^, household incoming^13^ and increased body mass index (BMI)^9,14^ were associated with prenatal stress or anxiety^15^. Moreover, maternal lifestyles during pregnancy, including eating disorders^5^, smoking and alcohol consumption^16,17^ and changes in social relationships^14,15^ also impacted the psychosocial characteristics of pregnant women. Increasing literatures to suggest a consensus that experience of adverse lifestyles can trigger stress or anxiety symptoms^6,15,16,18,19^. Finally, although information on risk factors for prenatal stress and anxiety is available in previous literatures, most studies only focused on one single risk factor.

As society develops, the lifestyles of maternal women have drastically changed. In the information rapid development day, the phone usage and working pace is increasing^20,21^ and the sleep quality is disturbing^22^. In some families, pregnant women have many burden, such as working, bring up babies, cooking and gestation. There are numerous studies reported that parts of new lifestyles was harmful to human physical health. Previous literatures have reported that using phone in the prenatal stage caused pathological changes in kidney tissues due to oxidative stress^23,24^, and that higher levels of electromagnetic radiation could lead to morphological changes in lymphocytes^25^. Moreover, animal experiment showed that extended electromagnetic radiation exposure induced oxidative stress in tissues of pregnant mice and their offspring^23,26^. Lack of sleep during pregnancy could have effect on endocrine system disorder, such as hypothalamic pituitary adrenal (HPA) axis abnormal response^27^. Always cooking would be more vulnerable to coronary heart disease^28^ and poor sleep quality^29^. We hypothesis these changed lifestyles would have effect on maternal prenatal psychological health. However, few studies are available on the influence of maternal new lifestyles on prenatal stress and anxiety, and simultaneously targeted the women with various new lifestyles on the risk of prenatal stress and anxiety.

Based on the above analysis, our study aimed to estimate whether maternal new lifestyle factors during pregnancy affected prenatal stress and anxiety in the Guangxi birth cohort study (GBCS), and to explore the associations between various lifestyles and antenatal stress and anxiety.

## Results

### Basic demographic characteristics

Descriptive analysis revealed that maternal age, pre-pregnancy BMI and gravidity history had significant statistical difference in sub-stress level groups. Inversely, other baseline characteristics had no significant differences in sub-stress groups and all of the demographic characteristics had no significant differences during anxiety and no anxiety groups (Table 1).

**Table 1:** Baseline characteristics of pregnant women with prenatal stress and anxiety.

Characteristics	Mild Stress	Moderate Stress	Severe Stress	*P* ^a^	Anxiety	No Anxiety	*P* ^a^
	n (%)	n (%)	n(%)		n (%)	n (%)	
N	286	801	404		119	1372	
Maternal age (Years)				0.000			0.068
≤24	48 (16.8)	175 (21.8)	177 (43.8)		34 (28.6)	300 (21.9)	
25∼29	106 (37.1)	362 (45.2)	111 (27.5)		47 (39.5)	598 (43.6)	
30∼34	85 (29.7)	176 (22.0)	91 (22.5)		32 (26.9)	320 (23.3)	
≥35	47 (16.4)	88 (11.0)	25 (6.2)		6 (5.0)	154 (11.2)	
Education (Years)				0.675			0.240
≤8	100 (35.0)	286 (35.7)	141 (34.9)		36 (30.3)	491 (35.8)	
8∼15	76 (26.6)	214 (26.7)	95 (23.5)		38 (31.9)	347 (25.3)	
≥15	110 (38.5)	301 (37.6)	168 (41.6)		45 (37.8)	534 (38.9)	
Pre-pregnancy BMI (Kg/cm^2^)				0.048			0.076
<18.5	57 (19.9)	218 (27.2)	102 (25.2)		35 (29.4)	342 (24.9)	
18.5∼23.9	188 (65.7)	496 (61.9)	258 (63.9)		69 (58.0)	873 (63.6)	
24.0∼27.9	38 (13.3)	67 (8.4)	36 (8.9)		9 (7.6)	132 (9.6)	
≥28.0	3 (1.0)	20 (2.5)	8 (2.0)		6 (5.0)	25 (1.8)	
Included Gestational age				0.956			0.065
First trimester	51 (17.8)	134 (16.7)	66 (16.3)		21 (17.6)	230 (16.8)	
Second trimester	204 (71.3)	589 (73.5)	298 (73.8)		79 (66.4)	1012 (73.8)	
Third trimester	31 (10.8)	78 (9.7)	40 (9.9)		19 (16.0)	130 (9.5)	
Gravidity history				0.001			0.331
First	86 (30.1)	299 (37.3)	178 (44.1)		40 (33.6)	523 (38.1)	
≥2	200 (69.9)	502 (62.7)	226 (55.9)		79 (66.4)	849 (61.9)	

BMI, Body Mass Index. ^a^*P* values were computed with the chi-square test.

### Selected maternal lifestyles

Comparisons between each stress group revealed that frequent cooking (χ^2^ = 9.943, *P* = 0.007), not having pets (χ^2^ = 10.782, P = 0.005), not having a baby at home (χ^2^ = 43.085, *P* < 0.001), a high level of phone usage per day (hours) (χ^2^ = 38.936, *P* < 0.001) and bad self-reported sleep quality (χ^2^ = 12.776, *P* = 0.012) were more likely to experience prenatal stress symptoms. Wearing radiation-proof clothing during pregnancy (χ^2^ = 5.378, *P* = 0.068) and having afternoon naps (χ^2^ = 3.029, *P* = 0.220) was not statistically different in different stress level groups (Table 2).

**Table 2:** Binary regression analysis of selected lifestyles and prenatal stress and anxiety.

Characteristics	Mild Stress	Moderate Stress	Severe Stress	*P* ^a^	Anxiety	No Anxiety	*P* ^a^
	n (%)	n (%)	n (%)		n (%)	n (%)	
N	286	801	404		119	1372	
Frequent Cooking				0.007			0.107
Yes	161 (56.3)	479 (59.8)	272 (67.3)		81 (68.1)	831 (60.6)	
No	125 (43.7)	322 (40.2)	132 (32.7)		38 (31.9)	541 (39.4)	
Having Pets				0.005			0.003
Yes	51 (17.83)	199 (24.84)	72 (17.82)		13 (10.92)	309 (22.52)	
No	235 (82.17)	602 (75.16)	332 (82.18)		106 (89.08)	1063 (77.48)	
Having babies at home				<0.001			0.132
None	128 (44.8)	466 (58.2)	280 (69.3)		77 (64.7)	797 (58.1)	
1	127 (44.4)	270 (33.7)	106 (26.2)		38 (31.9)	465 (33.9)	
≥2	31 (10.8)	65 (8.1)	18 (4.5)		4 (3.4)	110 (8.0)	
Phone usage time per day (hours)				<0.001			0.035
None	58 (20.28)	101 (12.61)	25 (6.19)		6 (5.04)	178 (12.97)	
<6	194 (67.83)	552 (68.91)	283 (70.05)		87 (73.11)	942 (68.66)	
≥6	34 (11.89)	147 (18.35)	94 (23.27)		26 (21.85)	252 (18.37)	
Wearing radiation-proof clothing				0.068			0.105
Yes	36 (12.59)	91 (11.36)	65 (16.09)		21 (17.65)	171 (12.46)	
No	250 (87.41)	710 (88.64)	339 (83.91)		98 (82.35)	1201 (87.54)	
Having afternoon nap				0.22			0.785
Yes	234 (81.82)	636 (79.40)	309 (76.49)		93 (78.15)	1086 (79.15)	
No	52 (18.18)	164 (20.60)	95 (23.51)		26 (21.85)	286 (20.85)	
Self-reported sleep quality				0.012			0.003
Good	120 (41.96)	290 (36.20)	127 (31.44)		34 (28.57)	505 (36.81)	
Generally	131 (45.80)	388 (48.44)	197 (48.76)		54 (45.38)	665 (48.47)	
Bad	35 (12.24)	116 (14.48)	80 (19.80)		31 (26.05)	202 (14.72)	

^a^*P* values were computed with the chi-square test.

For anxiety levels, not having pets (χ^2^ = 8.698, *P* = 0.003), increased phone usage per day (hours) (χ^2^ = 6.697, *P* = 0.035) and bad self-reported sleep quality (χ^2^ = 11.43, *P* = 0.003) was correlated to prenatal anxiety symptoms in two groups. Inversely, frequent cooking (χ^2^ = 2.592, *P* = 0.107), wearing radiation-proof clothing during pregnancy (χ^2^ = 2.622, *P* = 0.105), taking afternoon naps (χ^2^ = 0.075, *P* = 0.785) and having baby at home (χ^2^ = 4.047, *P* = 0.132) had no statistical differences in groups with and without anxiety (Table 2).

### The relationship between maternal lifestyles and prenatal stress

Ordinal logistic regression model analyses showed that less than 6 hours of phone usage per day (OR = 1.82, 95%CI: 1.32, 2.50) and more than 6 hours per day (OR = 2.43, 95%CI: 1.65, 3.58) and not having a baby at home (OR = 1.66, 95%CI: 1.05, 2.65) were positively correlated to prenatal stress, and positively correlated to an increased stress level, all *P* value was < 0.05 and all *P* trend of phone usage per day and having babies at home was < *0.01*. However, good or generally self-reported sleep quality (OR = 0.59, 95%CI: 0.44, 0.80 and OR = 0.72, 95%CI: 0.54, 0.96, respectively) and not frequent cooking (OR = 0.65, 95%CI: 0.53, 0.80) were negatively correlated to prenatal stress, and positively correlated to a decreased stress level, all *P* value was < 0.05 and *P* trend of self-reported sleep quality was <0.01. However, having pets was not correlated to prenatal stress (*P* > 0.05). The confounding factors including maternal age, pre-pregnant body mass index and gravidity history were adjusted in the ordinal logistic regression analysis (Table 3).

**Table 3:** Multivariate ordinal logistic regression: associations of selected lifestyles and prenatal stress.

Covariate	*β*	S.E	OR^a^ (95%CI)	*P*	*P trend*
Having babies at home					<0.01
≥2			1.00 (reference)		
1	0.01	0.21	1.01 (0.67,1.52)	0.966	
None	0.51	0.23	1.66 (1.05,2.65)	0.029	
Frequent cooking			1.00 (reference)		
Not frequent cooking	−0.44	0.11	0.65 (0.53,0.80)	<0.001	
Having pets			1.00 (reference)		
Not having pets	−0.11	0.13	0.89 (0.70,1.14)	0.370	
Phone usage time per day (hours)				<0.01	
None			1.00 (reference)		
<6	0.60	0.16	1.82 (1.32,2.50)	<0.001	
≥6	0.89	0.20	2.43 (1.65,3.58)	<0.001	
Self-reported sleep quality					<0.01
Bad			1.00 (reference)		
Generally	−0.33	0.15	0.72 (0.54,0.96)	0.023	
Good	−0.52	0.15	0.59 (0.44,0.80)	0.001	

*β*, standardised regression coefficients. S.E, standard error. OR, odds ratio. 95% CI, 95% confidence interval. ^a^ORs were adjusted for maternal age, pre-pregnancy Body Mass Index (BMI), gravidity history.

### The relationship between maternal lifestyles and prenatal anxiety

The results of binary logistic regression analyses examining the relationship between maternal lifestyles and prenatal anxiety are presented in Table 4. Participants who had less than 6 hours phone usage per day (OR = 2.42, 95%CI: 1.04, 5.65, *P* = 0.041) and more than 6 hours per day (OR = 2.62, 95%CI: 1.05, 6.55, *P* = 0.039) were almost twice as be likely to experience prenatal anxiety than participants with no phone usage, and were positively correlated to the level of prenatal anxiety, *P* trend was 0.016. Having pets (OR = 0.46, 95%CI: 0.26, 0.84, *P* = 0.011) and good or generally self-reported sleep quality (OR = 0.46, 95%CI: 0.27, 0.77, *P* = 0.003 and OR = 0.53, 95%CI: 0.33, 0.85, *P* = 0.008, respectively) were significantly associated with a decreased likelihood of prenatal anxiety, and associated with a decreased level of prenatal anxiety, *P* trend < 0.01. Maternal age and gravidity history were considered as confounding factors and adjusted in the logistic regression models (Table 4).

**Table 4:** Multivariate logistic regression: associations of selected lifestyles and prenatal anxiety.

Covariate	*β*	S.E	OR^a^ (95%CI)	*P*	*P trend*
Not having pets			1.00 (reference)		
Having pets	−0.77	0.30	0.46 (0.26, 0.84)	0.011	
Phone usage time per day (hours)					0.016
None			1.00 (reference)		
<6	0.88	0.43	2.42 (1.04, 5.65)	0.041	
≥6	0.96	0.47	2.62 (1.05, 6.55)	0.039	
Self-reported sleep quality					<0.01
Bad			1.00 (reference)		
Generally	−0.64	0.24	0.53 (0.33, 0.85)	0.008	
Good	−0.78	0.26	0.46 (0.27, 0.77)	0.003	

*β*, standardised regression coefficients. S.E, standard error. OR, odds ratio. 95% CI, 95% confidence interval. ^a^ORs were adjusted for maternal age, gravidity history.

## Discussion

From this cross-sectional study, we found that parts of maternal lifestyles during pregnancy had an impact on prenatal stress and anxiety. In particular, our results indicated that adverse lifestyles could increase the risk of stress and anxiety during pregnancy, and a variety of enjoyable activities and regular routines could relax pregnant women and decrease the risk of prenatal stress and anxiety. Our results showed that increased daily phone usage was a major risk factor in prenatal stress and anxiety. Moreover, good or generally self-reported sleep quality was negatively correlated to prenatal stress and anxiety, not frequent cooking and having pets was negatively correlated to prenatal stress and anxiety, respectively. Interestingly, we also found not having baby at home increase the risk of prenatal stress. Our findings are consistent with the findings of previous reports stating that adverse behaviour, such as prenatal or perinatal drinking^16,30,31^ or smoking^17^ could influence the psychology state of pregnant women. Moreover, our study revealed that prenatal mental health problems were prevalent in Guangxi. Indeed, all (100%) participants had elevated stress during their pregnancies and nearly a tenth of participants (7.98%) had elevated anxiety. However, we found the comorbidity of anxiety was lower than 12.43%, the international comorbidity of pregnant women having anxiety symptoms at various stages of pregnancy^6,32^. This could be because most of our participants were in their second trimester, previous studies have revealed that anxiety level are at their lowest in the second trimester^32^. Thus, we should pay more attention to antenatal stress and anxiety in pregnant women, and promote healthy lifestyles.

As the mobile internet develops, phone usage is rapid increasing and becoming a global problem^21^, which is very common in pregnant women^26^. Previous studies revealed that maternal women who more often used cell phone during pregnancy not only had an impact on maternal and new-born care practices^20,33^, but also lead to children behaviour abnormal at age seven^34^ and emotion and behaviour difficult at age eleven^35^. During all included variables, phone usage was the strongest risk factor for prenatal stress and anxiety. To our knowledge, this is the first study to report the association between phone usage during pregnancy and prenatal stress and anxiety. We also found that increased daily phone usage was positively correlated to an increased risk of prenatal stress and anxiety. If maternal women use phone >6 hours per day during pregnancy, the risk of prenatal stress and anxiety increased 2.43 and 2.62 times than not use phone, respectively. There are some explanations for our results: First, maternal women spend much time on mobile phone, limiting the time to do their daily work and resting^21^, which may influence the pregnant women’s mood. Second, most of pregnant women know that the electromagnetic radiation is harmful to adult’s and infant’s health^26^. It has been well documented that exposing to cell phone could lead to radio frequency electromagnetic field (RF-EMF)^36^. Moreover, Gao *et al*. found that mobile phone addiction (>4 hours/day) could increase the risk of anxiety of college students^21^. Either work requirement or addiction, more often using phone could increase the mood complexity of pregnant women.

In future pre-pregnancy health promotion, we should advise maternal women to limit the time spending on phone use, and broadcast excess RF- EMF exposure is harmful to human physical and mental health problems, fetuses or children would be more vulnerable to this potential influence, because neurological and organ systems is rapid development in early life and the extended exposure would be over the entire lifespan^37,38^.

Rushed lifestyles and gestational fatigue may lead to irregular sleep patterns, causing sleep deprivation, which can be harmful to pregnant women and infants^39–41^. Our study showed that pregnant women with poor sleep quality had higher levels of prenatal stress and anxiety, which was consistent with previous reports. In particular, previous studies reported that poor sleep quality was associated with prenatal depression^41,42^, stress^27,41^ and anxiety^27^. Okun *et al*.^41^ reported that pregnant women who sleep less than 7 hours per day may experience depressive symptoms. The biological mechanisms most associated with stress and anxiety was the joint stress-induced activation of the hypothalamic pituitary adrenal (HPA) axis and the progesterone (PROG) derived gamma-amino-butyric acid (GABA) ERGIC neurosteroids^27^, which has been implicated in reproductive mood disorders^43,44^. Previous studies suggested that maternal HPA axis responses to prenatal stress and anxiety were markedly suppressed in the second trimester^27^ and in late pregnancy^45,46^. Thus, it’s very important to promote the maternal women sleep quality during pregnancy, especially at the second and third trimester.

Household air pollution (HAP) arising from solid fuel use and meat cooking remains a global health threat^28,29^. Previous studies reported that exposure to HAP could have an impact on adverse birth outcomes, such as low birth weight^47,48^. To our knowledge, this is the first report focusing on the relationship between frequent cooking (≥3–5 times per week) during pregnancy and prenatal stress. Our results showed that not frequent cooking was negatively correlated to prenatal stress (OR = 0.65, 95%CI: 0.53, 0.80, *P* < 0.001). The majority of pregnant women know that cooking is one kind of household air pollution via the public platform and watching TV, which could increase maternal physical burden during pregnancy. Moreover, frequent cooking could increase the physically burden of pregnant women, especially who was with gestation reaction. In future pregnancy health care, we should advise pregnant women decrease the cooking times during pregnancy.

Interestingly, our study also showed that having pets during pregnancy was negatively correlated to prenatal anxiety (OR = 0.46, 95%CI: 0.26, 0.84, *P* = 0.011). To the best of our knowledge, this is the first study to investigate the relationship between having pets and maternal anxiety in pregnancy. The explanation for this results was that having pets could increase the life interesting and reduce the uncomfortableness of gestation. In addition, previous studies reported that exposure to pets, such as dogs or cats, could increase the resistance of immune system, and pregnant women could learn about this knowledge via internet, newspaper, *et al*. A meta-analysis reported that pregnant women exposed to household pets in the prenatal period were less likely to have an infant suffer from allergic diseases^49^. Havstad^50^ reported prenatal and postnatal pet exposure could result in lower IgE levels in very early life. Tun *et al*. found reduced streptococcal colonisation as a result of prenatal pet ownership may lower the risk of childhood metabolic and atopic disease^51^. With increasing reports of the beneficial effects of having pets at home, more and more households are getting pets. Although previous studies have revealed how beneficial pets are for pregnant women’s physical and psychological health, it is important to maintain a clean household and ensure all pets are up to date with their vaccinations.

Additionally, our study also discovered the relationship between having babies at home and prenatal stress, as far as we know, this is the first time this relationship has been studied by quantitative study. Our results showed that having babies at home could decrease the stress levels of maternal women, and there are no distinction during have one baby and ≥2 babies. These consisted with previous qualitative studies which have shown that women feel stressful if they are the first experience of motherhood^52^. First explanation for this is that first-time mothers were very concerned about the safety and wellbeing of their infants, and were very aware that they had responsibility for another life after delivery^53^. On the other hand, pregnant women who had no baby at home were concern about the new baby care after birth, because they are lack of confidence in themselves as new mothers to care for their baby. As a matter of fact, most women have very little opportunities to contact with babies and learn from others prior to becoming a mother^52^. At last, having a baby at home could increase the family’s happiness^53^. Thus, constant professional support during pregnancy is a perceived need for first- mother.

It is evident from our study that maternal lifestyles play an important role on prenatal stress and anxiety. It highlights the importance of pregnant women’s psychosocial health, assessing their risk factors and developing ways to improve their psychosocial resources to prevent antenatal stress and anxiety. Finally, our study also points to the need for greater research and clinical attention to antenatal stress and anxiety.

The key strength of our study as follow: First, to our knowledge, this is the first report of the relationship between phone usage, frequent cooking and having pets and prenatal maternal anxiety and stress. Second, this is the first report about the associations of various new lifestyles and prenatal stress and anxiety. In addition, there are several limitations in this study. In the cross-sectional study, prenatal stress and anxiety was not analysed in different trimesters because most participants were in their second trimester, which limited the study of prenatal stress and anxiety changes throughout the pregnancy. This will be considered in future studies. Second, because the data was collected from questionnaires, retrospective bias was inevitable. In future study, we could consider combine clinically diagnosed stress and anxiety disorders and self-reported symptoms. Moreover, we will increase the follow-up times to reduce the retrospective bias. In addition, we will do further studies because the evidences of reporting the associations of phone usage, frequent cooking, having pets and having babies at home and prenatal stress and anxiety are limited at present.

## Conclusions

From this study, we found that parts of maternal lifestyles play a vital role on prenatal stress and anxiety levels, indicating the importance of balanced maternal lifestyles during pregnancy. Moreover, the results could assist healthcare professionals in prevention, early identification and treatment of prenatal stress and anxiety.

## Materials and Methods

### Ethical approval and informed consent

The study was reviewed and approved by the Guangxi Medical University Medical Ethics Committee (ID: 2015(028)). All patients consented to participate in the research, and written informed consent was obtained from each patient.

### Study design and population

The cross-sectional study was based on the GBCS, an ongoing multicentre prospective cohort study in Guangxi, which aims to investigate pregnancy outcomes and the short and long-term health consequences of hereditary factors, environmental factors^54^ and psychological behavioural factors on children in the fast-paced society. 6,203 volunteers were recruited and followed-up from July 2015 until October 2016 in seven maternal & child health hospitals in Guangxi. In our present study, 1839 volunteers were recruited from GBCS at their prenatal examination between March and October in 2016. The inclusion criteria were the volunteers were between 18 and 45 years old, were born and lived in the study areas, had comprehensive ability to complete face-to-face interview questionnaires and had completed PSRS and SAS questionnaires. Excluding invalid questionnaires, 1547 were followed-up until they gave birth. After exclusions (abortion, stillbirth and birth defects), 1491 pregnant women were followed-up.

### Data Collection

Participants were invited to complete three questionnaires: the base line questionnaire, PSRS and SAS. The base line questionnaire was used to collect sociodemographic information and lifestyle information. The PSRS^55^ and SAS^56^ were performed to assess the anxiety and stress status of the participants.

### Basic demographic factors

Maternal age, ethnicity, education level, annual household income, profession, historic and present pregnancy information, such as whether they have regular menstruation or not and last menstrual period (LMP), gestational hypertension or gestational diabetes, *et al*. were collected using face-to-face questionnaires. Maternal and infant’s information was collected from the Guangxi Maternal and Child Health Information System.

### Lifestyles during pregnancy

Lifestyles, such as phone usage, hours of daily phone usage, whether the participant had afternoon naps, self-reported sleep quality, whether or not frequent cooking, having pets and having babies at home, *et al*. were collected from face-to-face questionnaire.

Phone usage was defined as maternal women use phone time per day during pregnancy which was collected from questionnaires by self-reported. It was divided into three groups, not using phone, using phone time <6 hours per day and ≥6 hours per day. Self-reported sleep quality was defined as maternal women’s sleep quality during pregnancy, which was also collected from questionnaires by self-reported and was recorded as “bad, generally and good”. Frequent cooking was defined as pregnant women cook ≥3–5 times per week during antenatal period. Having babies at home was defined as pregnant women have one or more babies at home, the age of them was from 1 to 12 years old. According to the number of babies, we divided it into “not having baby, having one baby and ≥2 babies” groups.

### PSRS

Antenatal stress was assessed with the PSRS^55^, a commonly used 30-item measure of stress in the antenatal period. The Chinese version was compiled and revised by Chen Chung-Hey and Pan Ying-Li and validated among pregnant women, with good psychometric properties^22,57^. This scale includes four stressors, including “Identify with a parent’s role”, “Concern about the health and safety of mother and children”, “Concern about the change of figure and activity” and “Else stressor”. PSRS is a 4- point Likert scale, from 0 (no stress) to 3 (severe stress). The total score is the mean of all items summed, with higher score indicating higher level of pregnancy stress. The total Cronbach’s α is 0.94.

### SAS

The SAS^56^ was chosen because it was specifically developed to measure maternal anxiety. It effectively evaluates the participant’s mood over the previous week. Many scholars have used this scale to assess the mental health of pregnant women^22,58,59^. SAS is composed of 20 items, a 5-point Likert scale, from 1 (None) to 4 (Most of the time). It is mainly used to assess the frequency of the symptoms mentioned in the items. The item score is standardised according to the formula: standardised score equals to integer (1.25 × item score)^59^. The standardised score is used as an index of prenatal anxiety, and the cut-off value is 50. The standardised scores 50 - 59, 60 - 69 and ≥70 present mild, moderate and severe anxiety, respectively. The Cronbach’s *α* is 0.91.

### Quality control

#### Investigations

All staff were master’s degree students of prophylactic medicine or clinical medicine. Before working, all investigators had completed unified training and simulated exercised. All of them were familiar with questionnaires and scales, understood the principles and announcements of the investigation and had mastered the unified methods and techniques required to guide subjects when they were completing the questionnaires.

After reviewing a great deal of literature, we modelled the research questionnaire on previous studies. The questionnaire was reviewed, modified and supplemented by relevant experts. Before the survey, we conducted pre-survey checks to discover all problems and revised the questionnaires accordingly.

#### Investigation process

We distributed the questionnaires and then collected them on time when participants completed. Investigators immediately checked and verified the integrity and validity of the questionnaires. To ensure the veracity of data, two independent workers completed the inputting process.

### Statistical analyses

All analyses were performed using SPSS version 21.0 (IBM). Frequencies were compared between groups by the χ^2^ test and were displayed as percentages (%). Ordinal multiple logistic regression analyses were used to examine the correlation between selected lifestyles and prenatal stress by calculating odds ratios (ORs) and their 95% confidence intervals (CIs). Binary logistic regression analyses were used to examine the association of selected lifestyles and prenatal anxiety, ORs and 95% CIs were also calculated. A *p* value of less than 0.05 was considered statistically significant.

### Data Availability

The datasets generated during and/or analysed during the current study are available from the corresponding author on reasonable request.
